# Disulfiram Eradicates Tumor-Initiating Hepatocellular Carcinoma Cells in ROS-p38 MAPK Pathway-Dependent and -Independent Manners

**DOI:** 10.1371/journal.pone.0084807

**Published:** 2014-01-13

**Authors:** Tetsuhiro Chiba, Eiichiro Suzuki, Kaori Yuki, Yoh Zen, Motohiko Oshima, Satoru Miyagi, Atsunori Saraya, Shuhei Koide, Tenyu Motoyama, Sadahisa Ogasawara, Yoshihiko Ooka, Akinobu Tawada, Tetsuya Nakatsura, Takehiro Hayashi, Taro Yamashita, Syuichi Kaneko, Masaru Miyazaki, Atsushi Iwama, Osamu Yokosuka

**Affiliations:** 1 Department of Gastroenterology and Nephrology, Graduate School of Medicine, Chiba University, Chiba, Japan; 2 Department of Cellular and Molecular Medicine, Graduate School of Medicine, Chiba University, Chiba, Japan; 3 Institute of Liver Studies, King's College Hospital, Denmark Hill, London, United Kingdom; 4 Division of Cancer Immunotherapy, Research Center for Innovative Oncology, National Cancer Center Hospital East, Kashiwa, Japan; 5 Department of Gastroenterology, Graduate School of Medicine, Kanazawa University, Kanazawa, Japan; 6 Department of General Surgery, Graduate School of Medicine, Chiba University, Chiba, Japan; University of Hong Kong, Hong Kong

## Abstract

Tumor-initiating cells (TICs) play a central role in tumor development, metastasis, and recurrence. In the present study, we investigated the effect of disulfiram (DSF), an inhibitor of aldehyde dehydrogenase, toward tumor-initiating hepatocellular carcinoma (HCC) cells. DSF treatment suppressed the anchorage-independent sphere formation of both HCC cells. Flow cytometric analyses showed that DSF but not 5-fluorouracil (5-FU) drastically reduces the number of tumor-initiating HCC cells. The sphere formation assays of epithelial cell adhesion molecule (EpCAM)^+^ HCC cells co-treated with p38-specific inhibitor revealed that DSF suppresses self-renewal capability mainly through the activation of reactive oxygen species (ROS)-p38 MAPK pathway. Microarray experiments also revealed the enrichment of the gene set involved in p38 MAPK signaling in EpCAM^+^ cells treated with DSF but not 5-FU. In addition, DSF appeared to downregulate *Glypican 3* (*GPC3*) in a manner independent of ROS-p38 MAPK pathway. GPC3 was co-expressed with EpCAM in HCC cell lines and primary HCC cells and *GPC3*-knockdown reduced the number of EpCAM^+^ cells by compromising their self-renewal capability and inducing the apoptosis. These results indicate that DSF impaired the tumorigenicity of tumor-initiating HCC cells through activation of ROS-p38 pathway and in part through the downregulation of *GPC3*. DSF might be a promising therapeutic agent for the eradication of tumor-initiating HCC cells.

## Introduction

Accumulating evidence has revealed that a minor population of tumor cells, called cancer stem cells or tumor-initiating cells (TICs), organizes a cellular hierarchy in a similar fashion to normal stem cells and shows pronounced tumorigenic activity in xenograft transplantations [1]. Recent progress in stem cell biology and technologies has contributed to the identification and characterization of TICs in various cancers including hepatocellular carcinoma (HCC) [2]. In HCC, side population cells and cells expressing several surface molecules such as epithelial cell adhesion molecule (EpCAM), CD133, CD90, and CD13 have been reported to function as TICs [3]. Besides the identification of tumor-initiating HCC cells, cancer-related molecules and signaling pathways, such as the polycomb group proteins, NANOG, AKT/PKB signal, and Wnt/β-catenin, have been shown to play an important role in maintaining or augmenting of tumor-initiating capability of TICs [4]. Although inhibitors of these molecules and signaling pathways may be potent TIC-targeting drugs, no effective therapy targeting TICs has been developed.

Disulfiram (DSF) is an irreversible inhibitor of aldehyde dehydrogenase and has been clinically used in the treatment of alcohol dependence for roughly 70 years [5]. DSF is a potent therapeutic agent in a wide range of human cancers. In addition, recent reports showed that DSF reduced the number of tumor-initiating cells and attenuated their sphere-forming abilities in breast cancer and glioblastoma [6], [7]. Although these findings indicate that DSF could eradicate TICs, the molecular machinery of its effect against TICs still remains largely unknown.

In the present study, we examined the effects of DSF on tumor-initiating HCC cells *in vitro* and *in vivo*. We found that DSF impaired their tumor-initiating ability and induced apoptosis by activating the reactive oxygen species (ROS)-p38 pathway. Furthermore, the downregulation of *Glypican3 (GPC3)* expression, which is caused independently of the ROS-p38 pathway, appeared to also be responsible for the anti-TIC effect of DSF.

## Results

### DSF inhibited tumorigenicity of HCC cells *in vitro* and in a xenograft transplantation model

**Figure 1:**
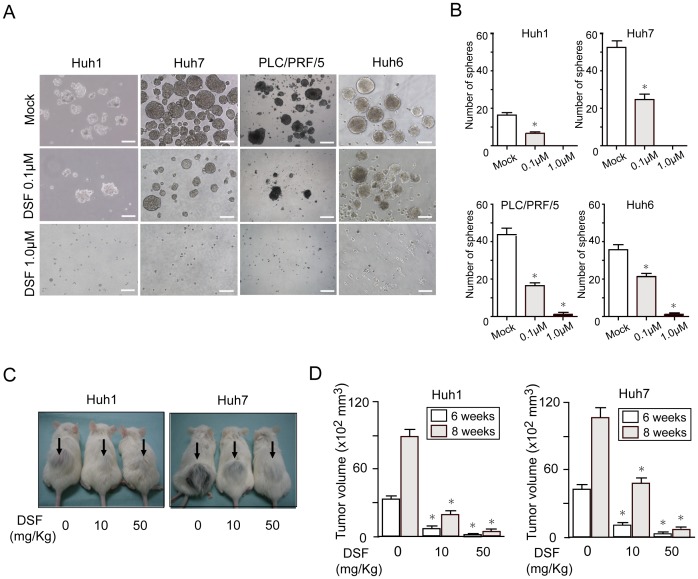
Sphere formation assays on HCC cells and xenograft transplantation. (A) Non-adherent sphere formation assay on HCC cell lines at day 14 of culture. Bright-field images are shown. Scale bar = 200 μm. (B) Number of large spheres generated from 1,000 HCC cells treated with DSF. *Statistically significant (p<0.05). (C) A total of 2×10^6^ Huh1 or Huh7 cells were transplanted into the subcutaneous space of NOD/SCID mice. The growth of subcutaneous tumors (arrows) was apparently suppressed by the DSF treatment in a dose-dependent manner 8 weeks after transplantation. (D) Subcutaneous tumor volume was determined 6 and 8 weeks after transplantation. *Statistically significant (p<0.05).

As shown in a variety of cancer cells [8]–[10], DSF treatment inhibited cell growth in both a time-dependent and dose-dependent manner in HCC cells (Figure S1A). Immunostaining of active caspase-3 (CASP3) showed that the DSF treatment induced apoptosis dose-dependently (Figure S1B). The percentage of apoptotic cells was roughly ten-fold higher among HCC cells treated with DSF (1 µM) than among control cells (Figure S1C). To examine whether DSF affected the tumorigenic ability of HCC cells, we conducted a non-adherent sphere assay, a standard assay for evaluating tumorigenic capacity. Sphere-forming ability was significantly impaired in DSF-treated HCC cell lines in a dose-dependent manner (Figure 1A and 1B). Subsequently, we determined the effects of DSF using a xenograft nonobese diabetic/severe combined immunodeficient (NOD/SCID) mouse model. After the implantation of 2×10^6^ Huh1 and Huh7 cells into NOD/SCID mice, DSF was administered intraperitoneally every other day. Tumor initiation and growth were apparently suppressed by the DSF treatment in a dose-dependent manner (Figure 1C and 1D). Together, these results indicate that DSF reduced the tumorigenicity of HCC cells.

### Loss-of-function assays of ALDH1 and ALDH2

DSF and its metabolites were shown to suppress ethanol metabolism mainly through the inhibition of cytosolic aldehyde dehydrogenase 1 (ALDH1) and mitochondrial ALDH2 [11]. It has been reported that *ALDH*-knockdown reduced proliferation and motility of lung cancer cells [12]. Because we previously showed that there was no association between the expression of ALDH1 and EpCAM or CD13 and that *ALDH1*-knockdown affected neither cell growth nor tumorigenicity in HCC cells [13], we conducted loss-of-function assays on ALDH2. We achieved the stable knockdown of *ALDH2* in Huh1 and Huh7 cells with lentivirus-mediated short hairpin RNA (shRNA) against *ALDH2* using enhanced red fluorescent protein (ERP) as a marker for infection (Figure S2A). No significant differences in cell growth and sphere formation were observed between *ALDH2*-knockdown cells and control cells expressing shRNA against *luciferase* (sh-*Luc*) (Figure S2B and S2C). Additionally, double-knockdown of *ALDH1* and *ALDH2* in the culture produced similar results to the single-knockdown of ALDH2 (Figure S2D-F). Taken together, the effects of DSF on HCC cells appeared to be independent of its inhibitory function toward ALDH1 and ALDH2.

### Decrease in the number of tumor-initiating HCC cells after DSF exposure

**Figure 2:**
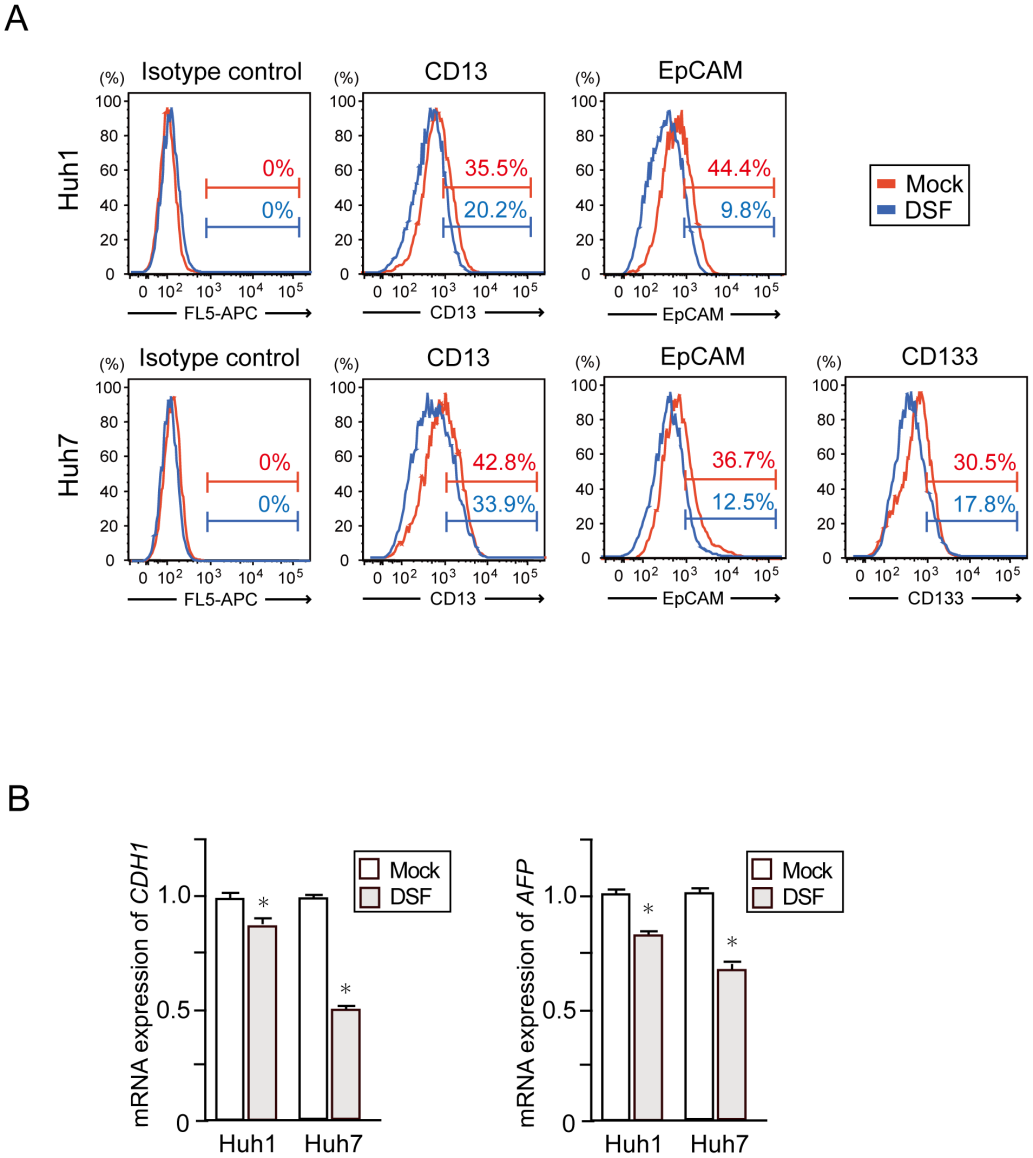
Flow cytometric analyses and quantitative RT-PCR analyses of HCC cells treated with DSF. (A) Flow cytometric profiles in Huh1 and Huh7 cells treated with DSF (0.1 μM) for 48 hours. The percentages of positive fractions for indicated markers are shown as the mean values for three independent analyses. (B) Real-time RT-PCR analyses of hepatic stem/progenitor cell marker genes. *Statistically significant (p<0.05).

We then examined the expression of various markers of tumor-initiating HCC cells such as CD13, epithelial cell adhesion molecule (EpCAM), and CD133 using flow cytometry. The DSF treatment appeared to decrease the number of HCC cells expressing these markers (Figure 2A). Among them, the EpCAM^high^ fraction markedly decreased from 44.4% to 9.8% in Huh1 cells and from 36.7% to 12.5% in Huh7 cells. Concordant with this, real-time RT-PCR analysis showed decreased expression of E-cadherin (CDH1) and alfa-fetoprotein (AFP), hepatic stem/progenitor cell markers, in DSF-treated cells (Figure 2B). In clear contrast, the 5-FU treatment resulted in the enrichment of TIC fractions (Figure S3). These results indicate that the biological effect of DSF differs from that of 5-FU, and is promising for the eradication of tumor-initiating HCC cells.

### DSF activated p38 MAPK in response to increased intracellular ROS levels in tumor-initiating HCC cells

**Figure 3:**
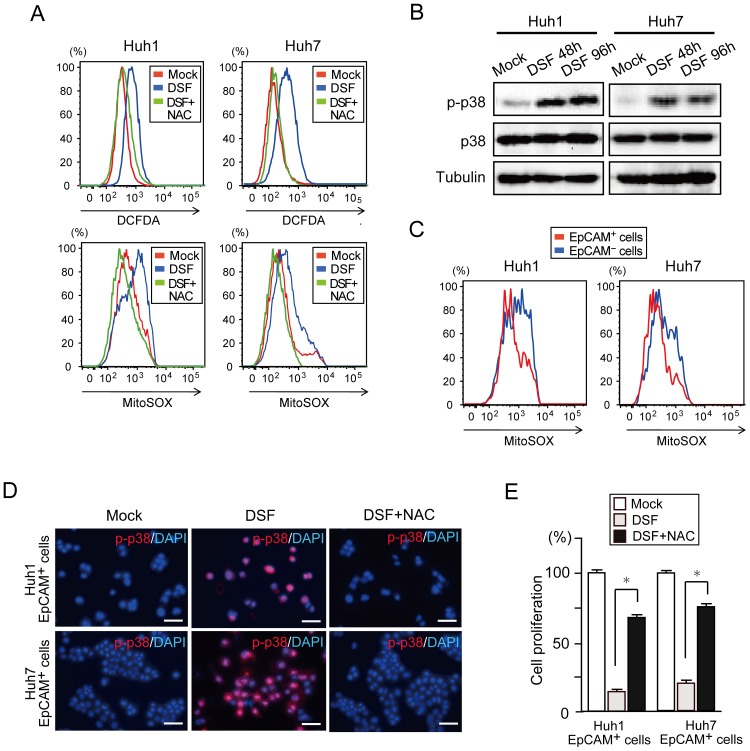
Activation of the ROS-p38 MAPK pathway in tumor-initiating EpCAM^+^ cells treated with DSF. (A) Flow cytometric analysis of ROS levels. Intracellular ROS concentrations were measured by DCFDA and MitoSOX staining. (B) Cells treated with DSF for 48 or 96 hours were subjected to Western blot analysis using phospho-p38 (p-p38), p38, and anti-tubulin (loading control) antibodies. (C) Flow cytometric analysis of ROS levels in view of EpCAM expression. Intracellular ROS concentrations were measured by MitoSOX staining. (D) Fluorescence images of EpCAM^+^ HCC cells. The expression of p-p38 (red) was merged with nuclear DAPI staining (blue). Scale bar = 100 μm. (E) Proliferation of EpCAM^+^ HCC cells at 96 hours in culture. The percentages of cells are shown. *Statistically significant (p<0.05).

Consistent with previous reports [6], [7], the present flow cytometric analyses showed that intracellular ROS levels were higher in DSF-treated HCC cells than in control cells (Figure 3A). However, co-treatment with NAC canceled this increase in ROS levels (Figure 3A). Western blotting showed increased levels of phosphorylated p38 after DSF exposure, which indicates p38 MAPK activation in HCC cells (Figure 3B). It has been well established that TICs maintain ROS at levels as low as normal stem cells [14], [15]. ROS levels were higher in EpCAM^−^ HCC cells than in EpCAM^+^ cells (Figure 3C). Notably, the co-treatment of sorted EpCAM^+^ cells with the antioxidant, NAC, canceled the phosphorylation of p38 induced by DSF (Figure 3D). Although EpCAM^−^ HCC cells generated only a small number of spheres, DSF treatment further reduced the number of spheres (Figure S4A and S4B). Approximately 90% of EpCAM^+^ cells treated with DSF was positive for phosphorylated p38 (Figure 3D), but the rate for EpCAM^−^ cells positive for phosphorylated p38 was nearly 25% (Figure S4C). The cell growth of EpCAM^+^ HCC cells was greatly restored by the additional NAC treatment (Figure 3E). Together, DSF caused activation of the ROS-p38 MAPK pathway in tumor-initiating HCC cells.

### p38 MAPK activation impaired self-renewal capability of tumor-initiating HCC cells

**Figure 4:**
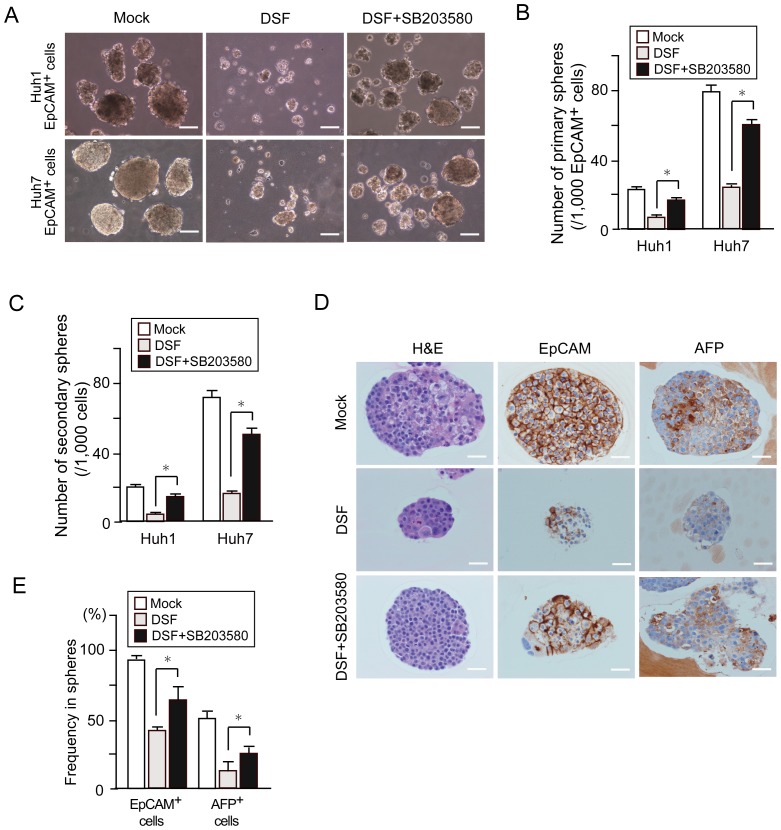
Sphere formation assays and immunocytochemical analyses in tumor-initiating EpCAM^+^ cells treated with a p38 inhibitor (SB203580). (A) Bright–field images of non-adherent spheres on day 14 of culture. Scale bar = 100 μm. (B) Number of large spheres derived from 1,000 EpCAM^+^ tumor cells on day 14 of culture. *Statistically significant (p<0.05). (C) Number of secondary spheres 14 days after replating. *Statistically significant (p<0.05). (D) H&E staining and immunocytochemical analysis of EpCAM and AFP in spheres derived from EpCAM^+^ cells. (E) Quantification of the percentage of EpCAM^+^ cells or AFP^+^ cells. *Statistically significant (p<0.05).

To examine the impact of p38 MAPK activation on tumor-initiating HCC cells, we conducted sphere formation assays on EpCAM^+^ HCC cells treated with DSF and/or SB203580, a specific inhibitor of p38 (Figure 4A). The co-treatment of cells with SB203580 largely abrogated the cell growth inhibition and apoptosis observed following the DSF treatment (Figure S5). Consistent with this, additional SB203580 treatment significantly restored the sphere-forming ability of EpCAM^+^ HCC cells (Figure 4B). Additionally, subsequent analyses for secondary sphere formation after replating showed results similar to those for the primary spheres (Figure 4C). These results indicate that activated p38 MAPK restricts the self-renewal of tumor-initiating HCC cells. We then conducted immunocytochemical analyses of the spheres and examined the expression of EpCAM and α-fetoprotein (AFP), a hepatic stem/progenitor cell marker [16]. Although the DSF treatment decreased the number of cells positive for AFP or EpCAM, co-treatment with DSF and SB203580 restored the number of positive cells (Figure 4D and 4E). Taken together, DSF impaired the tumor-initiating capability of HCC cells in part in a p38-dependent manner.

### Gene expression profiles of EpCAM^+^ HCC cells treated with DSF

**Figure 5:**
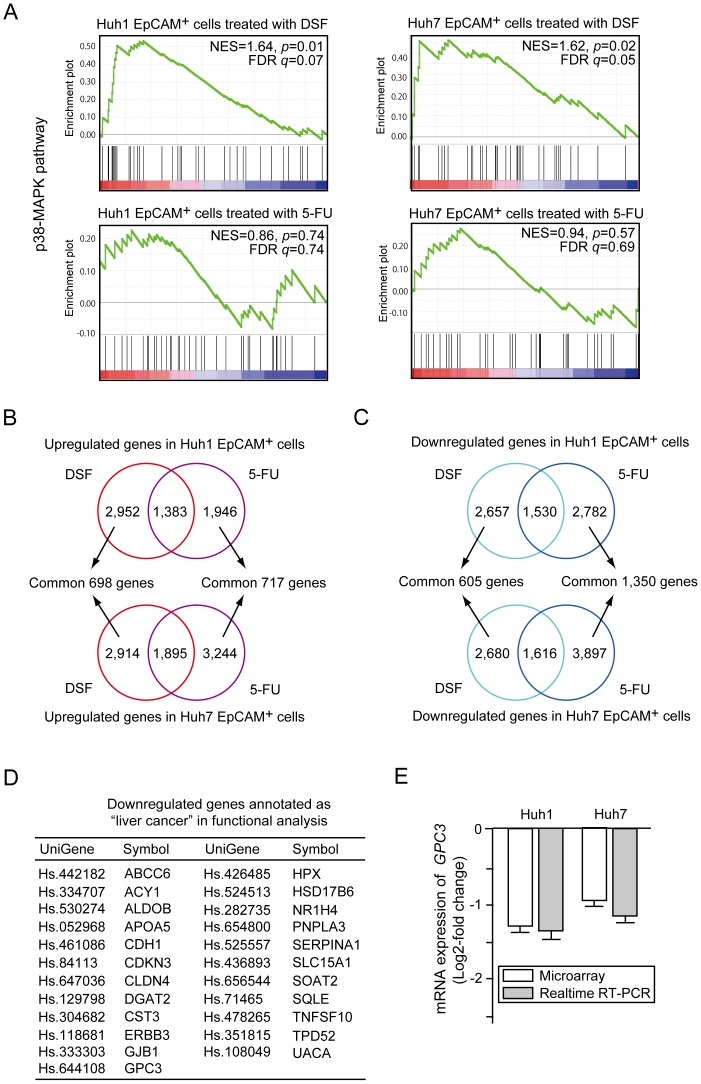
Gene expression profiles of EpCAM^+^ cells treated with DSF or 5-FU. (A) Gene set enrichment analysis (GSEA) of the p38-MAPK signaling pathway. Both the normalized enrichment score (NES) and false discovery rate (FDR) are shown in each enrichment plot. (B) Common upregulated genes in Huh1 cells (upper panel) and Huh7 cells (lower panel) after DSF or 5-FU treatment are depicted in Venn diagrams. (C) Common downregulated genes in Huh1 cells (upper panel) and Huh7 cells (lower panel) after DSF or 5-FU exposure are depicted in Venn diagrams. (D) A list of downregulated genes annotated as “liver cancer” in DSF-treated EpCAM^+^ HCC cells. (E) The expression of *GPC3* in DSF-treated EpCAM^+^ cells was compared to that in control cells. The data obtained by microarray analyses and quantitative RT-PCR analyses are presented.

EpCAM^+^ HCC cells treated with DSF or 5-FU for 48 hours were subjected to oligonucleotide microarray experiments. Concordant with the results presented in Figures 3 and 4, gene set enrichment analysis (GSEA) showed that EpCAM^+^ HCC cells treated with DSF, but not 5-FU were significantly enriched for genes involved in p38-MAPK signaling (Figure 5A) [17], [18]. The DSF treatment altered the expression of several genes involved in cell cycle regulation (Figure S6A and S6B). In particular, striking upregulation of *p57KIP2* was observed in Huh1 EpCAM^+^ cells. The gene set for the proteasome pathway showed a higher enrichment score in DSF-treated EpCAM^+^ HCC cells than in 5-FU-treated cells, although there was no significant difference (Figure S6C) [19].

We identified DSF-responsive genes (698 upregulated genes and 605 downregulated genes) and 5-FU-responsive genes (717 upregulated genes and 1,350 downregulated genes) (Figure 5B and 5C). Of interest, the DSF treatment causes no marked changes in the gene expression of the ROS scavenger pathway (Figure S6D). Furthermore, functional annotation analysis revealed different gene expression profiles between EpCAM^+^ HCC cells treated with DSF and 5-FU (Table S1 and S2). In particular, gene ontology terms enriched for downregulated genes were different. Additionally, 23 genes categorized into “liver cancer” were downregulated after exposure to DSF, but not 5-FU (Figure 5D). Among them, Glypican3 (GPC3) was shown to be specifically overexpressed in human HCC and *GPC3*-knockdown induced apoptosis in HCC cells [20], [21]. Quantitative RT-PCR showed that *GPC3* expression was downregulated in EpCAM^+^ HCC cells treated with DSF as shown in the microarray analyses (Figure 5E). However, the downregulation of *GPC3* was not observed in EpCAM^−^ HCC cells after DSF treatment (data not shown).

### Regulation of *GPC3* gene expression

To examine whether activation of the ROS-p38 MAPK pathway was crucial to the downregulation of *GPC3* expression by DSF, we examined *GPC3* expression in EpCAM^+^ HCC cells co-treated with NAC or SB203580. Neither NAC nor SB203580 restored the expression of *GPC3* (Figure S7A). In addition, proteasome inhibition by the MG132 treatment had no effect on *GPC3* expression (Figure S7B). These findings indicate that neither ROS-p38 MAPK pathway activation nor proteasome inhibition contributed to the downregulation of *GPC3* expression.

### Loss-of-function and gain-of-function assays of GPC3 in EpCAM^+^ HCC cells

**Figure 6:**
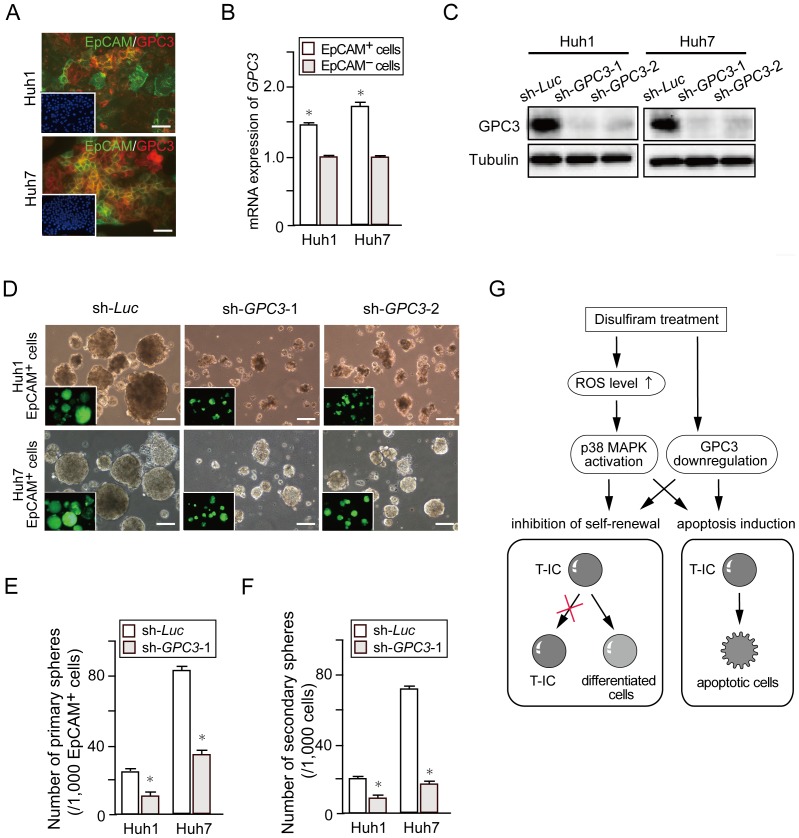
Impact of *GPC3* depletion on sorted EpCAM^+^ HCC cells. (A) Dual immunostaining was performed to detect the expression of EpCAM (green) and GPC3 (red). Nuclear DAPI staining is shown in the insets. Scale bar = 100 μm. (B) Real-time RT-PCR analysis of GPC3 expression in purified EpCAM^+^ cells. *Statistically significant (p<0.05). (C) Cells transduced with the indicated lentiviruses were subjected to Western blotting using anti-GPC3 and anti-tubulin (loading control) antibodies. (D) Bright–field images of non-adherent spheres on day 14 of culture. Fluorescence images are shown in the insets. Scale bar  = 100 μm. (E) Number of large spheres derived from 1,000 EpCAM^+^ or EpCAM^−^ cells at day 14 of culture. *Statistically significant (p<0.05). (F) Number of secondary spheres 14 days after replating. *Statistically significant (p<0.05). (G) A proposed model for the effect of DSF in targeting tumor-initiating HCC cells.

Dual immunostaining analyses showed that GPC3 and EpCAM were frequently co-expressed in HCC cells (Figure 6A). Moreover, quantitative RT-PCR revealed a higher level of GPC3 expression in the EpCAM^+^ fraction than in the EpCAM^−^ fraction (Figure 6B). Stable HCC cell lines expressing shRNA against *GPC3* or *luciferase* were successfully obtained by cell sorting with enhanced green fluorescent protein (EGFP) as a marker for viral infection. Western blot analysis of these cells showed that both shRNAs against *GPC3* (sh-*GPC3*-1 and sh-*GPC3*-2) markedly repressed GPC3 expression, although sh-*GPC3*-1 was more effective than sh-*GPC3*-2 (Figure 6C). *GPC3*-knockdown suppressed cell growth and induced apoptosis relative to sh-*Luc* (Figure S7C and S7D). Similarly, *GPC3*-knockdown markedly impaired primary sphere formation by EpCAM^+^ cells and EpCAM^−^ cells and more severely impaired secondary sphere formation (Figure 6D-F). Immunocytochemical analyses of the large spheres showed a decrease in the number of cells expressing AFP or EpCAM (Figure S7E and S7F). In contrast, the stable overexpression of *GPC3* promoted cell growth and sphere formation of tumor-initiating HCC cells (Figure S8). Together, these results indicate that *GPC3*-knockdown suppresses tumorigenicity of HCC cells by directly affecting the cell growth and the self-renewal of TIC.

## Discussion

High levels of ALDH activity are characteristic of normal stem cells in a variety of organs. The human ALDH superfamily consists of 19 putatively functional genes [22]. ALDH1 is a major isoform in mammalian tissues and functions as a stem cell marker in liver and mammary stem cells [23], [24]. Recent reports have indicated ALDH1 to be a useful marker for the enrichment of TICs from various cell lines and primary tumors. It has been shown that a high level of ALDH1 expression correlates with malignant phenotypes and an unfavorable prognosis in a range of cancers [24].

In this study, we first showed that DSF inhibited the proliferation and sphere-forming ability of HCC cells in a dose-dependent manner. In addition, DSF suppressed tumor growth in xenograft transplant experiments using NOD/SCID mice. Our flow cytometric analysis showed that the DSF treatment caused a significant decrease in the number of tumor-initiating HCC cells expressing surface markers such as CD13, CD133, and EpCAM. Knockdown of *ALDH1* and *ALDH2* in HCC cells had no effect on cell proliferation and sphere-forming ability in the culture. Our findings suggest that DSF exerts its anti-HCC function in an ALDH-independent fashion.

HSCs have been shown to tightly control intracellular ROS levels to maintain long-term self-renewal and survival [25]. Conversely, activation of p38 MAPK upon an elevation in ROS levels resulted in the exhaustion of HSCs [26]. Similarly, TICs in a wide range of tumors exhibited lower concentrations of ROS than corresponding non-TICs. In addition, lower ROS levels in TICs were shown to be closely associated with both chemo-sensitivity and radio-sensitivity [15]. In the present study, we confirmed that EpCAM^+^ HCC cells contained lower ROS levels than EpCAM^−^ cells. Because previous studies reported that DSF activated the ROS-p38 MAPK pathway and thereby suppressed the sphere-forming ability of TICs [6], [7], we examined whether exposure to DSF activated the ROS-p38 MAPK pathway in tumor-initiating HCC cells. As expected, the treatment of EpCAM^+^ HCC cells with NAC canceled p38 activation. Moreover, the SB203580 treatment largely restored the tumorigenicity of EpCAM^+^ HCC cells. These findings indicate that the ROS-p38 MAPK pathway is directly associated with cell growth and tumor-initiating capability of HCC cells. Low levels of ROS in TICs have been attributable to the activation of the ROS scavenger pathway [27]. The present microarray results showed comparatively high expression levels of ROS scavenger genes such as *GCLM* and *GSS* in purified EpCAM^+^ HCC cells. However, the DSF treatment caused no marked changes to the ROS scavenger genes. Considering that not only H2DCFDA staining but also MitoSOX staining showed a high ROS level in DSF-treated EpCAM^+^ HCC cells, DSF might increases mitochondrial ROS production rather than impairs the scavenging of ROS. Further analysis is required to clarify this point.

Of interest, our microarray analyses revealed that DSF acted in a manner different from 5-FU. The GSEA results support the present biological finfings and implicate the activation of p38 in the anti-TIC activity of DSF. Importantly, the 23 genes in the “liver cancer” category were significantly downregulated after the DSF exposure, but none of them was significantly altered after the 5-FU treatment. One of these genes, GPC3, was frequently overexpressed in HCC and increased GPC3 expression was correlated with a poor prognosis among HCC patients [20], [21]. A clinical trial using a GPC3 peptide vaccine in patients with advanced HCC has also been carried out [28]. While GPC3 functions as a marker for normal hepatic stem/progenitor cells [29], the immunostaining analyses showed an association between the expression of EpCAM and GPC3 in both HCC cell lines and HCC surgical specimens (data not shown) and the higher basal expression of GPC3 in EpCAM^+^ cells than EpCAM^−^ cells. Lentiviral knockdown of *GPC3* significantly reduced the sphere-forming ability of EpCAM^+^ HCC cells. Additionally, replating assays and immunocytochemical analyses of EpCAM and AFP indicated that GPC3 regulated tumor-initiating HCC cells. Although it appears that DSF suppresses the tumorigenicity of tumor-initiating HCC cells in part by downregulating GPC3 expression, further analyses would be of importance to clarify the mechanisms underlying the downregulation of *GPC3* by DSF.

Finally, our findings successfully demonstrated that DSF significantly reduced the number of tumor-initiating HCC cells through apoptosis induction and the conversion to non-TICs. These effects appeared to be attributable to the activation of the ROS-p38 MAPK pathway and gene silencing with GPC3 (Figure 6G). Further analyses of the genes listed here are necessary to determine the effects of DSF. Recent reports showed that TICs of brain tumors reside in vascular niches in which endothelial cells maintain the TICs in an undifferentiated state [30]. Bevacizumab, a vascular endothelial growth factor (VEGF)-specific inhibitor, causes a drastic decrease in the number of TICs in vascular niches by inhibiting the self-renewal of TICs [31]. Although the niche for TICs in HCC remains to be elucidated, combination therapy using DSF and the anti-angiogenic multi-kinase inhibitor sorafenib might be effective in the eradication of tumor-initiating HCC cells.

## Materials and Methods

### Ethics statement

All experiments using the mice were performed in accordance with our institutional guidelines for the use of laboratory animals and approved by the review board for animal experiments of Chiba University (approval ID: 22–187).

### Mice

Nonobese diabetic/severe combined immunodeficiency (NOD/SCID) mice (Sankyo-Lab Service, Tsukuba, Japan) were bred and maintained in accordance with our institutional guidelines for the use of laboratory animals.

### Cell culture and reagents

The HCC cell lines were obtained from the Health Science Research Resources Bank (HSRRB, Osaka, Japan). DSF was kindly provided by Mitsubishi Tanabe Pharma Corporation. Cells were treated with DSF/CuCl_2_ (0.1 or l µM) or 5-FU (1 µM; Sigma-Aldrich, St Louis, MO). Cells were treated with MG132 (10 µM, Cayman Chemical, Ann Arbor, MI), *N*-Acetyl-_L_-cysteine (NAC) (10 µM, Sigma), and SB203860 (10 mM, Sigma).

### Non-adherent sphere culture

For the sphere formation assay of Huh1, Huh6 and Huh7 cells, 1,000 cells were plated onto ultra-low attachment 6-well plates (Corning, Corning, NY). For the assay of PLC/PRF/5 cells, 500 cells were plated onto NanoCulture 24-well plates (Scivax, Kawasaki, Japan). The number of spheres (>100 µm in diameter) was counted on day 14 of culture. For the secondary sphere formation, a single cell suspension derived from primary colonies was obtained using a Neurocult chemical dissociation kit (StemCell Technologies, Vancouver, BC). Paraffin-embedded sections of the spheres were subjected to hematoxylin & eosin (H&E) staining and immunohistochemical staining with anti-EpCAM (Cell Signaling Technology, Beverly, MA) and anti-AFP (Dako Cytomation, Carpinteria, CA) antibodies.

### Cell sorting and analysis

Single-cell suspensions were stained with allophycocyanin (APC)-conjugated anti-EpCAM antibody and anti-CD13 antibody (Biolegend, San Diego, CA) or APC-conjugated anti-CD133/1 antibody (Miltenyi Biotec, Auburn, CA). After the incubation, 1 µg/ml of propidium iodide was added to eliminate dead cells. Flow cytometirc cell sorting and analyses were performed using FACSAria or FACSCanto (BD Biosciences, San Jose, CA). Intracellular ROS levels were determined by flow cytometry using H2DCFDA (Sigma) and MitoSOX (Molecular Probes, Eugene, OR) staining.

### Xenograft transplantation using NOD/SCID mice

A total of 2×10^6^ Huh1 and Huh7 cells were suspended in DMEM and Matrigel (BD) (1∶1). The cells were implanted into the subcutaneous space of the backs of NOD/SCID mice. DSF (10 or 50 mg/Kg) was administered intraperitoneally every other day.

### Western blotting

DSF-treated HCC cells were subjected to Western blot analysis using anti-p38 (Santa Cruz Biotechnology, Santa Cruz, CA), anti-phospho-p38 (Cell Signaling Technology), and anti-tubulin (Oncogene Science, Cambridge, MA) antibodies. *ALDH2*-knockdown cells and *ALDH1*-and *ALDH2*-double knockdown cells were subjected to Western blotting using anti-ALDH1 (BD Biosciences) and anti-ALDH2 (Abcam, Cambridge, MA) antibodies. *GPC3*-knockdown cells selected by cell sorting for enhanced green fluorescent protein (EGFP) expression were also subjected to Western blot analysis using anti-GPC3 antibody (Santa Cruz Biotechnology).

### Lentiviral production and transduction

A lentiviral vector carrying ERP (CS-H1-shRNA-RfA-ERP) expressing shRNAs against *ALDH2* (target sequence: sh-*ALDH2*-1, 5′-GCCCACTGTGTTTGGAGATGT-3′; sh-*ALDH2*-2, 5′-GCTGTCTTCACAAAGGATTTG-3′) was constructed for the double knockdown of *ALDH1* and *ALDH2*. Lentiviral vectors (CS-H1-shRNA-EF-1a-EGFP) expressing shRNAs against murine GPC3 (target sequence: sh-*GPC3*-1, 5′-GGCTCTGAATCTTGGAATTGA-3′; sh-*GPC3*-2, 5′-GGGACTGATGATGGTTAAACC-3′) were also constructed. Recombinant lentiviruses were produced as described elsewhere [32].

### Generation of stable GPC3-expressing cells

Human GPC3 cDNA was cloned into a site upstream of IRES-neomycin in the pLP-IRESneo vector (Clontech, Palo. Alto, CA). Stable transfection into Huh1 cells with G418 selection was performed.

### Reverse transcription-polymerase chain reaction (RT-PCR)

Quantitative RT-PCR was performed with an ABI PRISM 7300 Sequence Detection System (Applied Biosystems) using the Universal Probe Library System (Roche Diagnostics) according to the manufacturer's directions. The sequences of primers are listed in Table S3. Relative quantification was conducted by using the comparative cycle threshold (Ct) method.

### Immunocytochemistry

After fixation with 2% paraformaldehyde and blocking in 10% goat serum, the cells were stained with anti-EpCAM (Cell Signaling Technology) and anti-phospho-p38 MAPK (Cell Signaling Technology) antibodies. Subsequently, the cells were incubated with Alexa-488–conjugated goat anti-mouse immunoglobulin G (IgG) (Molecular Probes) and Alexa-555–conjugated goat anti-rabbit IgG (Molecular Probes). The cells were coverslipped using a mounting medium containing 4′, 6-diamidino-2-phenylindole dihydrochloride (DAPI) (Vector Laboratories, Burlingame, CA). For detection of apoptosis, the cells were also stained with an anti-active caspase-3 (CASP3) antibody (Chemicon, Temecula, CA), followed by incubation with Alexa-555 conjugated goat anti-rabbit IgG (Molecular Probes).

### Microarray analysis

Cy3-labeled complementary RNA was hybridized to a SurePrint G3 Human GE 8×60 K microarray (Agilent Technologies, Santa Clara, CA). Array images were scanned using a DNA Microarray Scanner (Agilent) and analyzed using Feature Extraction version 10.27.1.1. (Agilent). Normalization was performed using GeneSpring GX11.5.1 (Agilent). The expression value (Signal) for each probe set was calculated using GeneSpring GX 12.0 (Agilent). Data were obtained for triplicate samples from three independent experiments. The data were subjected to normalization using GeneSpring normalization algorithms (Agilent). Only gene expression levels with statistical significance (p<0.05) were recorded as being “detected” above background levels, and genes with expression levels below this statistical threshold were considered “absent.” To identify differentially expressed genes in EpCAM^+^ cells, we selected probe sets that exhibited gene expression changes with statistical significance as follows: (i) genes exhibiting a change greater than 1.5-fold (p<0.05), (ii) genes exhibiting a change from 1.0 to 1.5-fold (p<0.01), and (iii) switch-on type (upregulated from the “absent” to “present” level) and switch-off type genes (downregulated from the “present” to “absent” level) exhibiting a change greater than 4.0-fold (p<0.01). Moreover, functional analyses were performed using Ingenuity Pathway Analysis (IPA) version 12402621 (Ingenuity Systems). To identify gene signatures after DSF or 5-FU treatment, gene set enrichment analysis (GSEA) was also conducted [33]. The raw data are available at http://www.ncbi.nlm.nih.gov/geo/(accession number; GSE 42318).

### Statistical analysis

Data are presented as the mean ± SEM. Statistical differences between 2 groups were analyzed using the Mann-Whitney U test. P values less than 0.05 were considered significant.

## Supporting Information

Figure S1 *In vitro* assays of HCC cells treated with DSF. (A) Dose-dependent and time-dependent inhibition of proliferation in HCC cells treated with DSF. *Statistically significant (p<0.05). (B) Detection of apoptotic cell death by immunostaining for active CASP3. Nuclear DAPI staining is shown in the insets. Scale bar = 100 μm. (C) Quantification of the percentage of apoptotic cells. *Statistically significant (p<0.05).
(TIF)

Figure S2 In vitro assay for *ALDH2*-knockdown and double knockdown of *ALDH1* and *ALDH2*. (A) Cells transduced with the indicated lentiviruses were subjected to Western blotting using anti-*ALDH2* and anti-tubulin (loading control) antibodies. (B) Cell proliferation in *ALDH2*-knockdown HCC cells was monitored by counting cell numbers. (C) Number of primary spheres generated from 1,000 cells at day 14 of culture. (D) Cells co-transduced with the indicated lentiviruses were subjected to Western blotting using anti-*ALDH1* antibody, anti-*ALDH2* and anti-tubulin (loading control) antibodies. (E) Bright-field (upper panels) images of non-adherent spheres at day 14 of culture. Scale bar = 100 μm. EGFP and RFP expression in double-knockdown spheres are shown in the insets. (F) Number of primary spheres generated from 1,000 cells at day 14 of culture.
(TIF)

Figure S3 Flow cytometric analyses of HCC cells treated with 5-FU. Flow cytometric profiles in cells treated with 5-FU (10μg/ml) for 48 hours. The percentages of positive fractions for the indicated markers are shown as the mean values for three independent analyses.
(TIF)

Figure S4 In vitro assay of sorted EpCAM^−^ cells treated with DSF. (A) Non-adherent sphere formation assay on EpCAM^−^ cells at day 14 of culture. Bright-field images are shown. Scale bar = 200 μm. (B) Number of large spheres generated from 1,000 HCC cells treated with DSF. *Statistically significant (p<0.05). (C) Fluorescence images of EpCAM^−^ HCC cells. The expression of p-p38 (red) was merged with nuclear DAPI staining (blue). Scale bar = 100 μm.
(TIF)

Figure S5 In vitro assay of sorted EpCAM^+^ cells co-treated with DSF and a p38-specific inhibitor (SB203580). (A) Cell proliferation at 96 hours in culture. *Statistically significant (p<0.05). (B) Quantification of apoptotic cells based on the results of immunostaining for CASP3. *Statistically significant (p<0.05).
(TIF)

Figure S6 Gene expression profiles of EpCAM^+^ cells treated with DSF or 5-FU. (A) Log2-fold heat map of genes involved in cell cycle in EpCAM^+^ cells treated with DSF. (B) Quantitative RT-PCR analyses of cell cycle-related genes. *Statistically significant (p<0.05). (C) Gene set enrichment analysis (GSEA) of the proteasome pathway in EpCAM^+^ cells treated with DSF or 5-FU. Both the normalized enrichment score (NES) and false discovery rate (FDR) are shown in each enrichment plot. (D) Log2-fold heat map of genes involved in the ROS scavenger pathway in EpCAM^+^ cells treated with DSF or 5-FU.
(TIF)

Figure S7 Regulatory machinery of *GPC3* expression and loss-of-function assay of GPC3 in tumor-initiating HCC cells. (A) Quantitative RT-PCR analyses of *GPC3* expression in EpCAM^+^ HCC cells co-treated with DSF and NAC or SB203580. *Statistically significant (p<0.05). (B) Quantitative RT-PCR analyses of *GPC3* expression in EpCAM^+^ HCC cells treated with MG132. (C) Cell proliferation in *GPC3*-knockdown HCC cells at 96 hours in culture. *Statistically significant (p<0.05). (D) Quantification of apoptosis in cells transduced with indicated the lentiviruses based on the results of immunostaining for CASP3. *Statistically significant (p<0.05). (E) H&E staining and immunocytochemical analysis of EpCAM and AFP in spheres derived from EpCAM^+^ cells. Scale bar = 20 μm. (F) Quantification of the percentage of EpCAM^+^ or AFP^+^ cells. *Statistically significant (p<0.05).
(TIF)

Figure S8 Gain-of-function assay of GPC3 in Huh1 EpCAM^+^ cells. (A) Cells transduced with the indicated retroviruses were subjected to Western blotting using anti-GPC3 and anti-tubulin (loading control) antibodies. (B) Proliferation of Huh1 EpCAM^+^ cells at 96 hours in culture. The percentages of cells are shown. *Statistically significant (p<0.05). (C) Bright–field images of Huh1 EpCAM^+^ cells in non-adherent sphere formation at day 14 of culture. Scale bar = 100 μm. (D) Number of large spheres derived from 1,000 EpCAM^+^ cells on day 14 of culture. *Statistically significant (p<0.05). (E) Number of secondary spheres 14 days after replating. *Statistically significant (p<0.05).
(TIF)

Table S1 Top five ontology terms with molecular and cellular function of upregulated genes after DSF or 5-FU treatment.
(DOC)

Table S2 Top five ontology terms with molecular and cellular function of downregulated genes after DSF or 5-FU treatment.
(DOC)

Table S3 Primer sequences used for real-time RT-PCR.
(DOC)

## References

[1] Jordan CT, Guzman ML, Noble M (2006) Cancer stem cells. N Engl J Med 355: 1253–1261. 16990388 10.1056/NEJMra061808

[2] Visvader JE, Lindeman GJ (2012) Cancer stem cells: current status and evolving complexities. Cell Stem Cell 10: 717–728. 22704512 10.1016/j.stem.2012.05.007

[3] Ji J, Wang XW (2012) Clinical implications of cancer stem cell biology in hepatocellular carcinoma. Semin Oncol 39: 461–472. 22846863 10.1053/j.seminoncol.2012.05.011 PMC3409471

[4] Rountree CB, Mishra L, Willenbring H (2012) Stem cells in liver disease and cancer: Recent advances on the path to new therapies. Hepatology 55: 298–306. 22030746 10.1002/hep.24762 PMC3245372

[5] Chen D, Cui QC, Yang H, Dou QP (2006) Disulfiram, a clinically used anti-alcoholism drug and copper-binding agent, induces apoptotic cell death in breast cancer cultures and xenografts via inhibition of the proteasome activity. Cancer Res 66: 10425–10433. 17079463 10.1158/0008-5472.CAN-06-2126

[6] Yip NC, Fombon IS, Liu P, Brown S, Kannappan V, et al (2011) Disulfiram modulated ROS-MAPK and NFκB pathways and targeted breast cancer cells with cancer stem cell-like properties. Br J Cancer 104: 1564–1574. 21487404 10.1038/bjc.2011.126 PMC3101904

[7] Liu P, Brown S, Goktug T, Channathodiyil P, Kannappan V, et al (2012) Cytotoxic effect of disulfiram/copper on human glioblastoma cell lines and ALDH-positive cancer-stem-like cells. Br J Cancer 107: 1488–1497. 23033007 10.1038/bjc.2012.442 PMC3493777

[8] Cen D, Gonzalez RI, Buckmeier JA, Kahlon RS, Tohidian NB, et al (2002) Disulfiram induces apoptosis in human melanoma cells: a redox-related process. Mol Cancer Ther 1: 197–204. 12467214

[9] Wang W, McLeod HL, Cassidy J (2003) Disulfiram-mediated inhibition of NF-kappaB activity enhances cytotoxicity of 5-fluorouracil in human colorectal cancer cell lines. Int J Cancer 104: 504–11. 12584750 10.1002/ijc.10972

[10] Zhang H, Chen D, Ringler J, Chen W, Cui QC, et al (2010) Disulfiram treatment facilitates phosphoinositide 3-kinase inhibition in human breast cancer cells in vitro and in vivo. Cancer Res 70: 3996–4004. 20424113 10.1158/0008-5472.CAN-09-3752 PMC3827685

[11] Moreb JS, Baker HV, Chang LJ, Amaya M, Lopez MC, et al (2008) ALDH isozymes downregulation affects cell growth, cell motility and gene expression in lung cancer cells. Mol Cancer 7: 87. 19025616 10.1186/1476-4598-7-87 PMC2605459

[12] Johansson B (1992) A review of the pharmacokinetics and pharmacodynamics of disulfiram and its metabolites. Acta Psychiatr Scand Suppl 369: 15–26. 10.1111/j.1600-0447.1992.tb03310.x 1471547

[13] Suzuki E, Chiba T, Zen Y, Miyagi S, Tada M, et al (2012) Aldehyde dehydrogenase 1 is associated with recurrence-free survival but not stem cell-like properties in hepatocellular carcinoma. Hepatol Res 42: 1100–1111. 22583771 10.1111/j.1872-034X.2012.01028.x

[14] Ito K, Hirao A, Arai F, Matsuoka S, Takubo K, et al (2004) Regulation of oxidative stress by ATM is required for self-renewal of hematopoietic stem cells. Nature 431: 997–1002. 15496926 10.1038/nature02989

[15] Diehn M, Cho RW, Lobo NA, Kalisky T, Dorie MJ, et al (2009) Association of reactive oxygen species levels and radioresistance in cancer stem cells. Nature 458: 780–783. 19194462 10.1038/nature07733 PMC2778612

[16] Yamashita T, Forgues M, Wang W, Kim JW, Ye Q, et al (2008) EpCAM and alpha-fetoprotein expression defines novel prognostic subtypes of hepatocellular carcinoma. Cancer Res 2008 68: 1451–1461. 10.1158/0008-5472.CAN-07-6013 18316609

[17] Schaefer CF, Anthony K, Krupa S, Buchoff J, Day M, et al.. (2009) PID: the Pathway Interaction Database. Nucleic Acids Res 37(Database issue): D674–679. 10.1093/nar/gkn653 PMC2686461 18832364

[18] Science Signaling Web Site. Available: http://stke.sciencemag.org/cgi/cm/stkecmCMP_10958 Accessed 2012 January 3.

[19] Wong DJ, Nuyten DS, Regev A, Lin M, Adler AS, et al (2008) Revealing targeted therapy for human cancer by gene module maps. Cancer Res 68: 369–378. 18199530 10.1158/0008-5472.CAN-07-0382

[20] Midorikawa Y, Ishikawa S, Iwanari H, Imamura T, Sakamoto H, et al (2003) Glypican-3, overexpressed in hepatocellular carcinoma, modulates FGF2 and BMP-7 signaling. Int J Cancer 103: 455–465. 12478660 10.1002/ijc.10856

[21] Liu S, Li Y, Chen W, Zheng P, Liu T, et al (2012) Silencing glypican-3 expression induces apoptosis in human hepatocellular carcinoma cells. Biochem Biophys Res Commun 419: 656–661. 22382024 10.1016/j.bbrc.2012.02.069

[22] Marchitti SA, Brocker C, Stagos D, Vasiliou V (2008) Non-P450 aldehyde oxidizing enzymes: the aldehyde dehydrogenase superfamily. Expert Opin Drug Metab Toxicol 4: 697–720. 18611112 10.1517/17425250802102627 PMC2658643

[23] Dollé L, Best J, Empsen C, Mei J, Van Rossen E, et al (2012) Successful isolation of liver progenitor cells by aldehyde dehydrogenase activity in naïve mice. Hepatology 55: 540–552. 21953779 10.1002/hep.24693

[24] Ginestier C, Hur MH, Charafe-Jauffret E, Monville F, Dutcher J, et al (2007) ALDH1 is a marker of normal and malignant human mammary stem cells and a predictor of poor clinical outcome. Cell Stem Cell 1: 555–567. 18371393 10.1016/j.stem.2007.08.014 PMC2423808

[25] Tothova Z, Kollipara R, Huntly BJ, Lee BH, Castrillon DH, et al (2007) FoxOs are critical mediators of hematopoietic stem cell resistance to physiologic oxidative stress. 128: 325–339. 10.1016/j.cell.2007.01.003 17254970

[26] Ito K, Hirao A, Arai F, Takubo K, Matsuoka S, et al (2006) Reactive oxygen species act through p38 MAPK to limit the lifespan of hematopoietic stem cells. Nat Med 12: 446–451. 16565722 10.1038/nm1388

[27] Ishimoto T, Nagano O, Yae T, Tamada M, Motohara T, et al (2011) CD44 variant regulates redox status in cancer cells by stabilizing the xCT subunit of system xc- and thereby promotes tumor growth. Cancer Cell 19: 387–400. 21397861 10.1016/j.ccr.2011.01.038

[28] Sawada Y, Yoshikawa T, Nobuoka D, Shirakawa H, Kuronuma T, et al (2012) Phase I trial of a glypican-3-derived peptide vaccine for advanced hepatocellular carcinoma: immunologic evidence and potential for improving overall survival. Clin Cancer Res 18: 3686–3696. 22577059 10.1158/1078-0432.CCR-11-3044

[29] Grozdanov PN, Yovchev MI, Dabeva MD (2006) The oncofetal protein glypican-3 is a novel marker of hepatic progenitor/oval cells. Lab Invest 86: 1272–1284. 17117158 10.1038/labinvest.3700479

[30] Gilbertson RJ, Rich JN (2007) Making a tumour's bed: glioblastoma stem cells and the vascular niche. Nat Rev Cancer 7: 733–736. 17882276 10.1038/nrc2246

[31] Calabrese C, Poppleton H, Kocak M, Hogg TL, Fuller C, et al (2007) A perivascular niche for brain tumor stem cells. Cancer Cell 11: 69–82. 17222791 10.1016/j.ccr.2006.11.020

[32] Iwama A, Oguro H, Negishi M, Kato Y, Morita Y, et al (2004) Enhanced self-renewal of hematopoietic stem cells mediated by the polycomb gene product Bmi-1. Immunity 21: 843–851. 15589172 10.1016/j.immuni.2004.11.004

[33] Subramanian A, Tamayo P, Mootha VK, Mukherjee S, Ebert BL, et al (2005) Gene set enrichment analysis: a knowledge-based approach for interpreting genome-wide expression profiles. Proc Natl Acad Sci U S A 102: 15545–15550. 16199517 10.1073/pnas.0506580102 PMC1239896

