# Supplementary figures and images for: Disulfiram Eradicates Tumor-Initiating Hepatocellular Carcinoma Cells in ROS-p38 MAPK Pathway-Dependent and -Independent Manners
Source: PLoS One. 2014 Jan 13;9(1):e84807. doi: 10.1371/journal.pone.0084807 (PMC3890271; doi:10.1371/journal.pone.0084807)

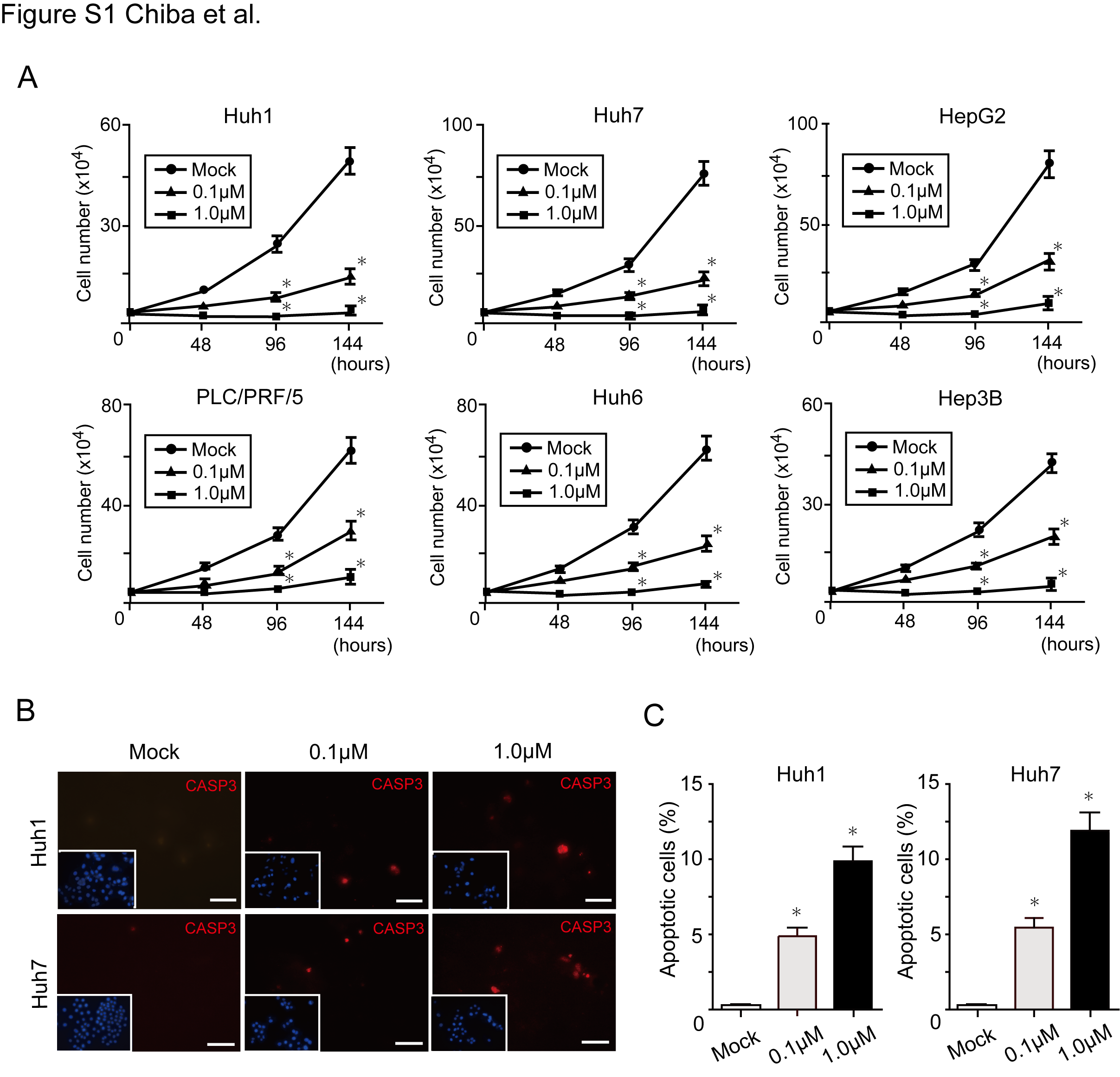

Supplement: Figure S1 — In vitro assays of HCC cells treated with DSF. (A) Dose-dependent and time-dependent inhibition of proliferation in HCC cells treated with DSF. *Statistically significant (p<0.05). (B) Detection of apoptotic cell death by immunostaining for active CASP3. Nuclear DAPI staining is shown in the insets. Scale bar = 100 μm. (C) Quantification of the percentage of apoptotic cells. *Statistically significant (p<0.05). (TIF) [file pone.0084807.s001.tif]

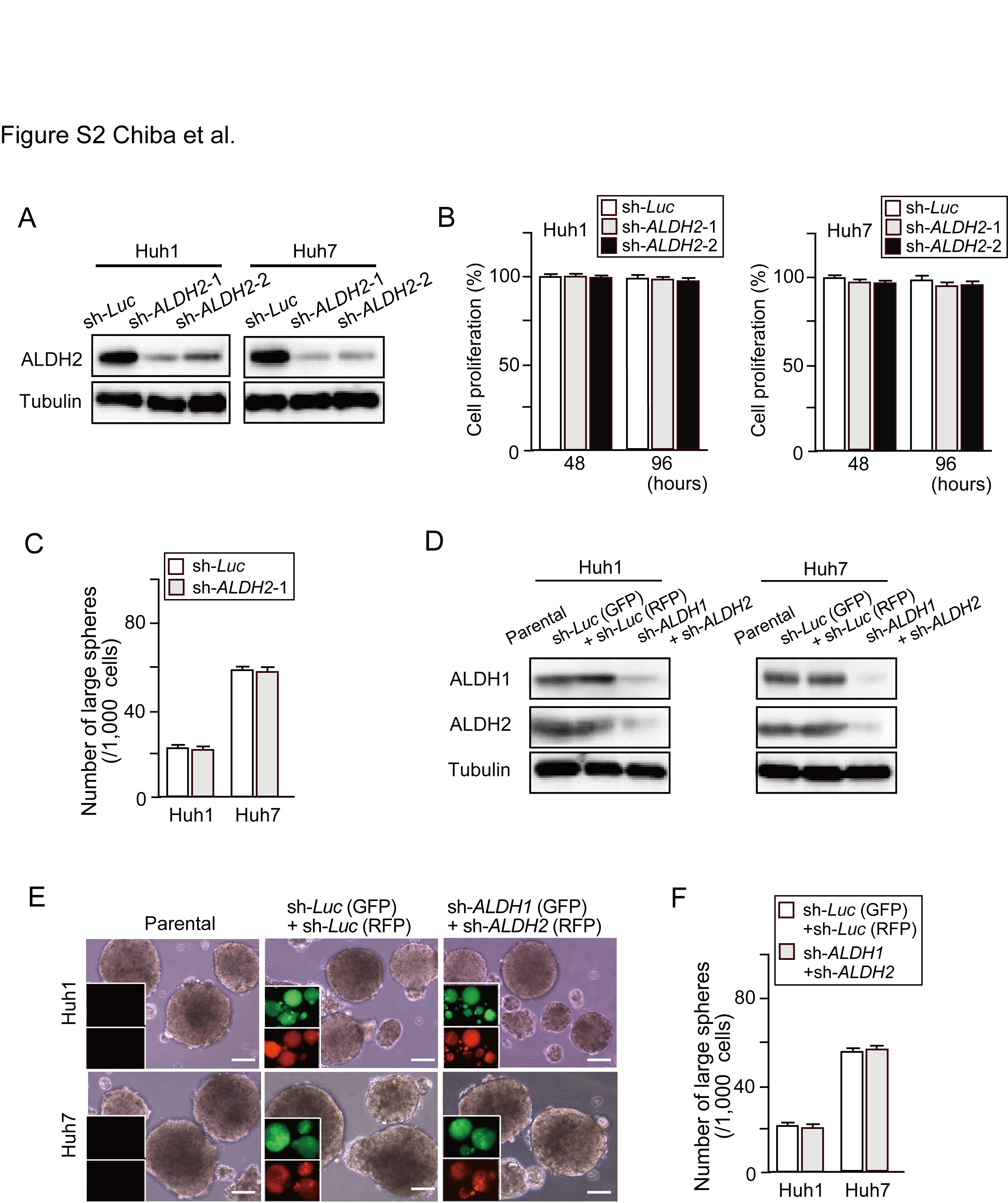

Supplement: Figure S2 — In vitro assay for ALDH2-knockdown and double knockdown of ALDH1 and ALDH2. (A) Cells transduced with the indicated lentiviruses were subjected to Western blotting using anti-ALDH2 and anti-tubulin (loading control) antibodies. (B) Cell proliferation in ALDH2-knockdown HCC cells was monitored by counting cell numbers. (C) Number of primary spheres generated from 1,000 cells at day 14 of culture. (D) Cells co-transduced with the indicated lentiviruses were subjected to Western blotting using anti-ALDH1 antibody, anti-ALDH2 and anti-tubulin (loading control) antibodies. (E) Bright-field (upper panels) images of non-adherent spheres at day 14 of culture. Scale bar = 100 μm. EGFP and RFP expression in double-knockdown spheres are shown in the insets. (F) Number of primary spheres generated from 1,000 cells at day 14 of culture. (TIF) [file pone.0084807.s002.tif]

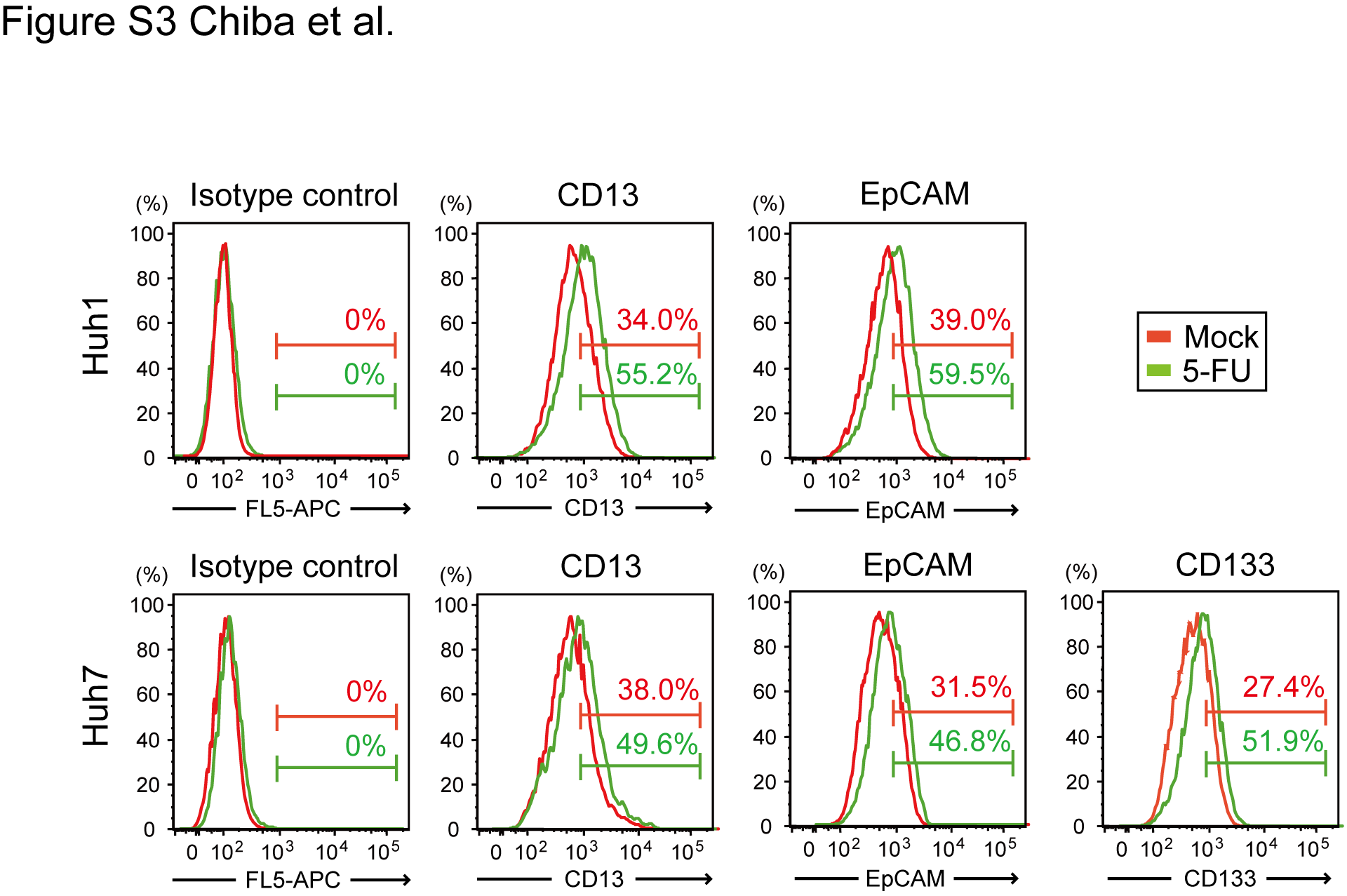

Supplement: Figure S3 — Flow cytometric analyses of HCC cells treated with 5-FU. Flow cytometric profiles in cells treated with 5-FU (10μg/ml) for 48 hours. The percentages of positive fractions for the indicated markers are shown as the mean values for three independent analyses. (TIF) [file pone.0084807.s003.tif]

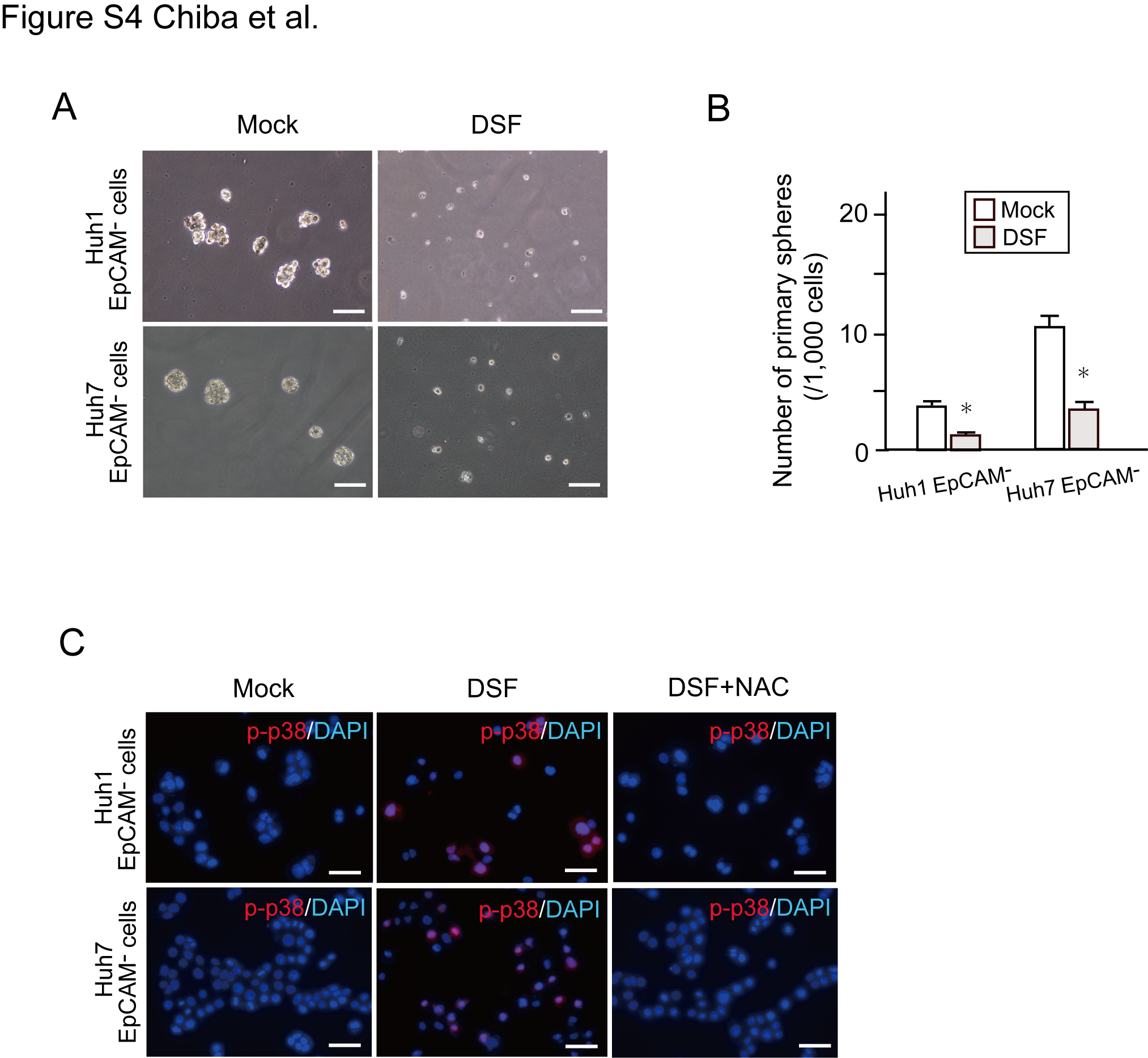

Supplement: Figure S4 — In vitro assay of sorted EpCAM− cells treated with DSF. (A) Non-adherent sphere formation assay on EpCAM− cells at day 14 of culture. Bright-field images are shown. Scale bar = 200 μm. (B) Number of large spheres generated from 1,000 HCC cells treated with DSF. *Statistically significant (p<0.05). (C) Fluorescence images of EpCAM− HCC cells. The expression of p-p38 (red) was merged with nuclear DAPI staining (blue). Scale bar = 100 μm. (TIF) [file pone.0084807.s004.tif]

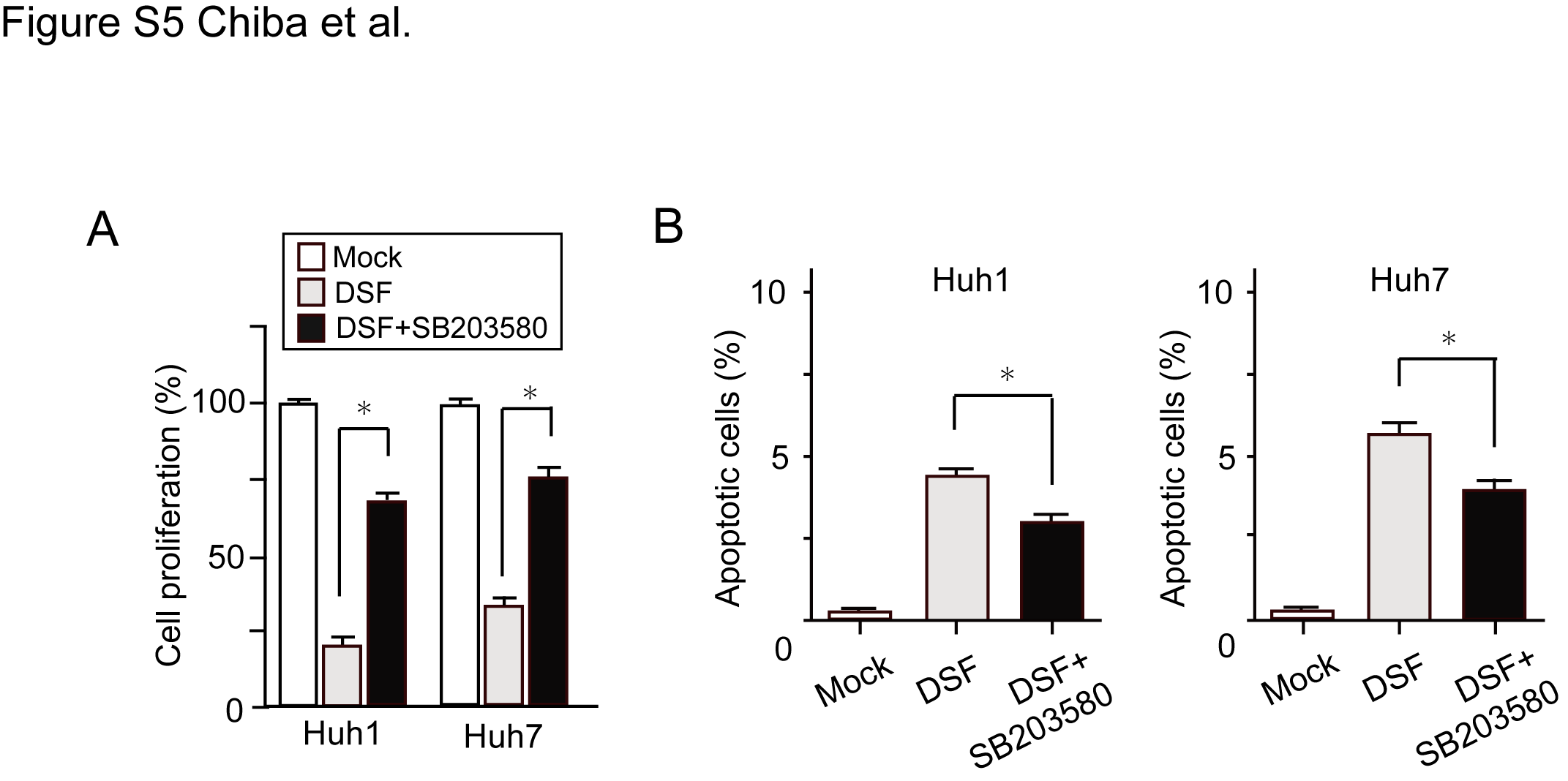

Supplement: Figure S5 — In vitro assay of sorted EpCAM+ cells co-treated with DSF and a p38-specific inhibitor (SB203580). (A) Cell proliferation at 96 hours in culture. *Statistically significant (p<0.05). (B) Quantification of apoptotic cells based on the results of immunostaining for CASP3. *Statistically significant (p<0.05). (TIF) [file pone.0084807.s005.tif]

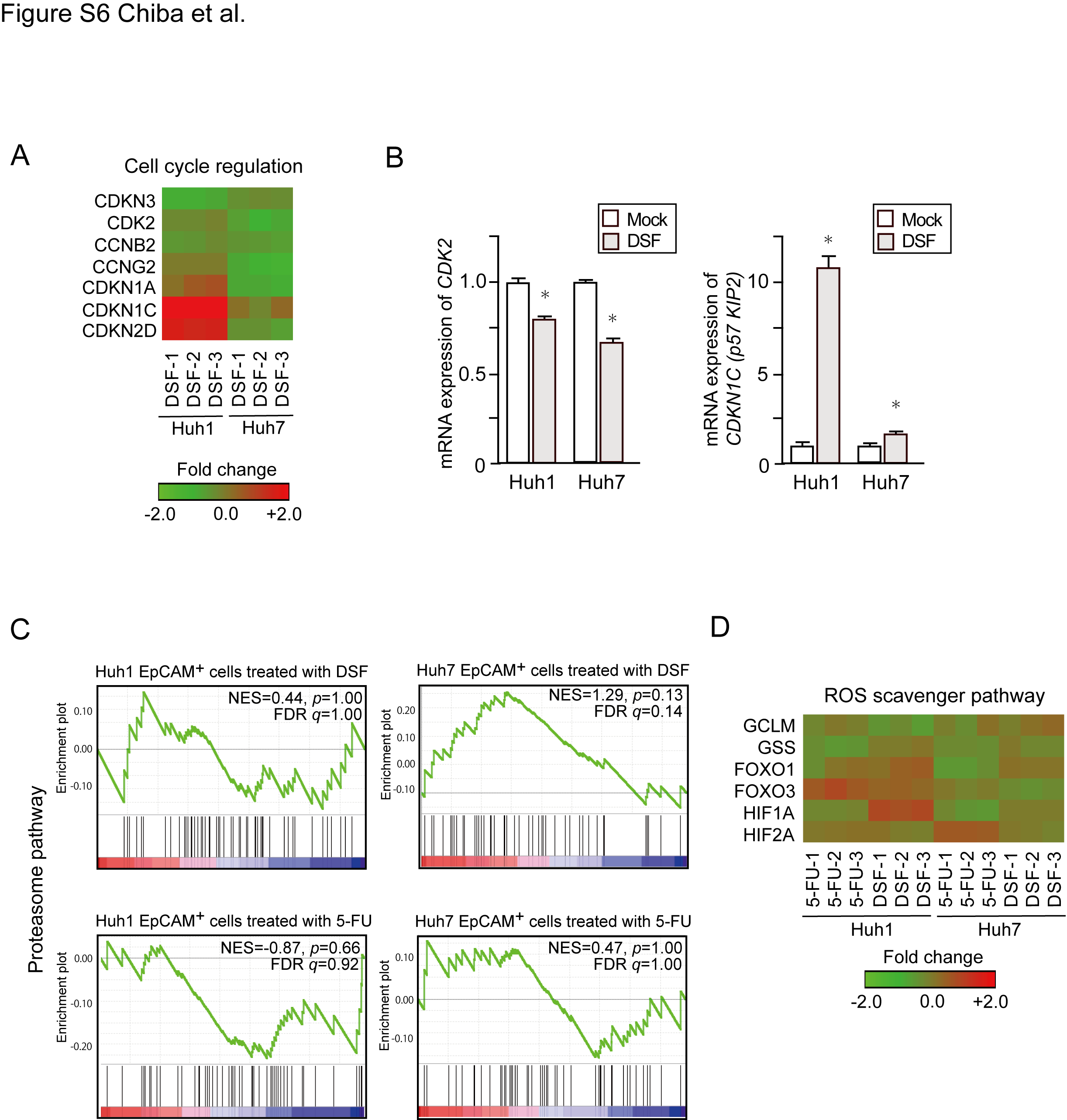

Supplement: Figure S6 — Gene expression profiles of EpCAM+ cells treated with DSF or 5-FU. (A) Log2-fold heat map of genes involved in cell cycle in EpCAM+ cells treated with DSF. (B) Quantitative RT-PCR analyses of cell cycle-related genes. *Statistically significant (p<0.05). (C) Gene set enrichment analysis (GSEA) of the proteasome pathway in EpCAM+ cells treated with DSF or 5-FU. Both the normalized enrichment score (NES) and false discovery rate (FDR) are shown in each enrichment plot. (D) Log2-fold heat map of genes involved in the ROS scavenger pathway in EpCAM+ cells treated with DSF or 5-FU. (TIF) [file pone.0084807.s006.tif]

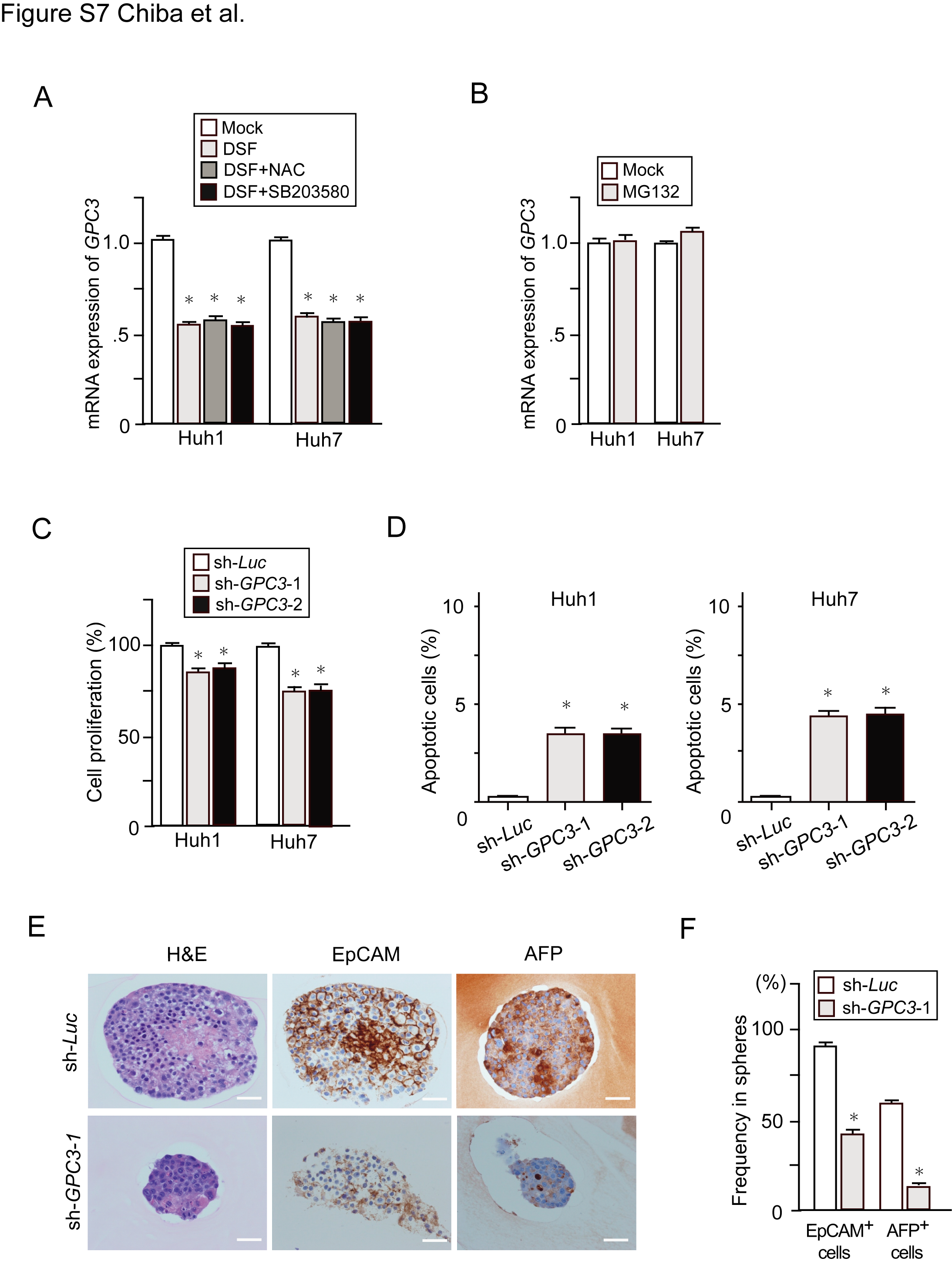

Supplement: Figure S7 — Regulatory machinery of GPC3 expression and loss-of-function assay of GPC3 in tumor-initiating HCC cells. (A) Quantitative RT-PCR analyses of GPC3 expression in EpCAM+ HCC cells co-treated with DSF and NAC or SB203580. *Statistically significant (p<0.05). (B) Quantitative RT-PCR analyses of GPC3 expression in EpCAM+ HCC cells treated with MG132. (C) Cell proliferation in GPC3-knockdown HCC cells at 96 hours in culture. *Statistically significant (p<0.05). (D) Quantification of apoptosis in cells transduced with indicated the lentiviruses based on the results of immunostaining for CASP3. *Statistically significant (p<0.05). (E) H&E staining and immunocytochemical analysis of EpCAM and AFP in spheres derived from EpCAM+ cells. Scale bar = 20 μm. (F) Quantification of the percentage of EpCAM+ or AFP+ cells. *Statistically significant (p<0.05). (TIF) [file pone.0084807.s007.tif]

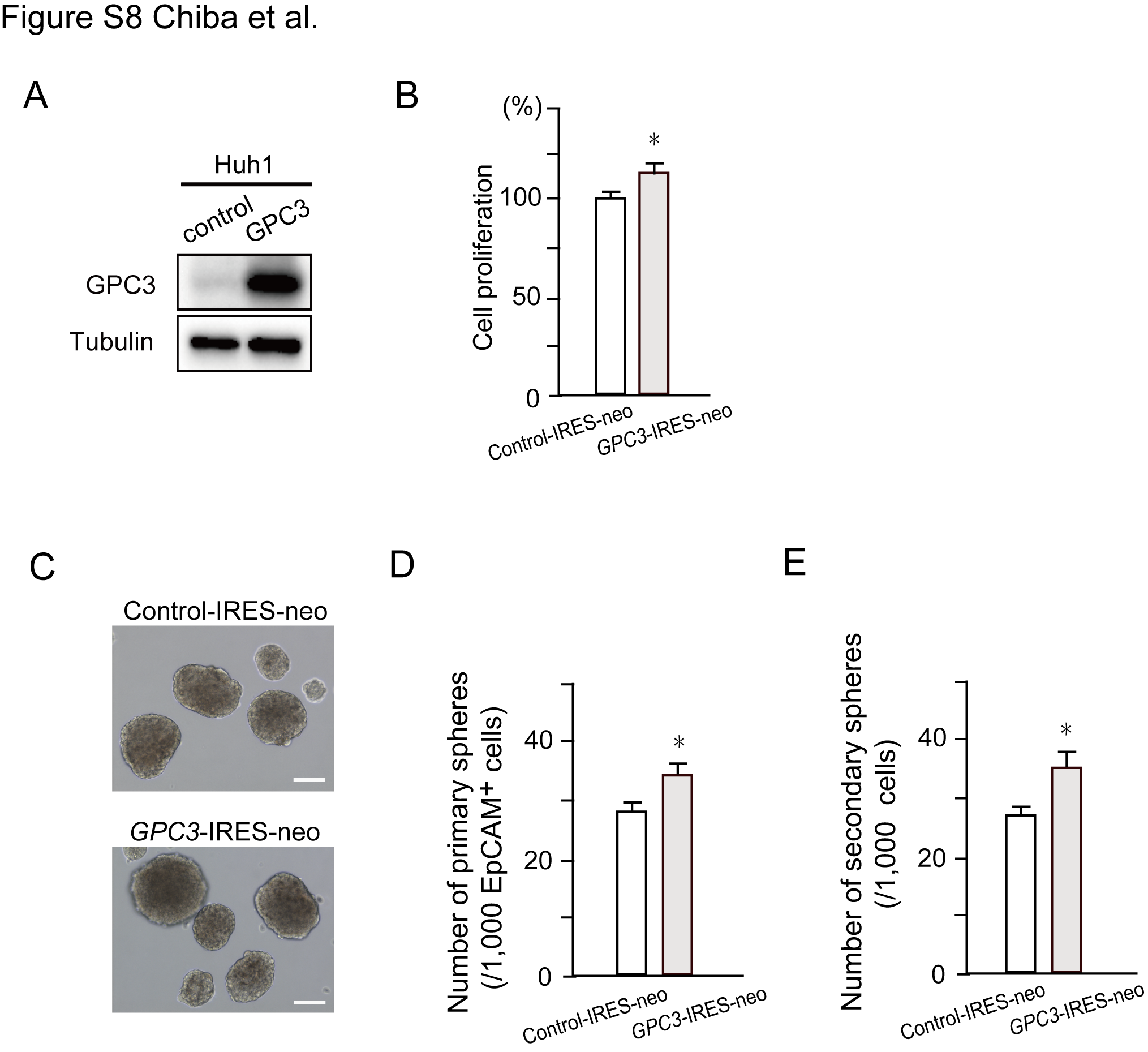

Supplement: Figure S8 — Gain-of-function assay of GPC3 in Huh1 EpCAM+ cells. (A) Cells transduced with the indicated retroviruses were subjected to Western blotting using anti-GPC3 and anti-tubulin (loading control) antibodies. (B) Proliferation of Huh1 EpCAM+ cells at 96 hours in culture. The percentages of cells are shown. *Statistically significant (p<0.05). (C) Bright–field images of Huh1 EpCAM+ cells in non-adherent sphere formation at day 14 of culture. Scale bar = 100 μm. (D) Number of large spheres derived from 1,000 EpCAM+ cells on day 14 of culture. *Statistically significant (p<0.05). (E) Number of secondary spheres 14 days after replating. *Statistically significant (p<0.05). (TIF) [file pone.0084807.s008.tif]
