# Supplementary material for: Disulfiram Eradicates Tumor-Initiating Hepatocellular Carcinoma Cells in ROS-p38 MAPK Pathway-Dependent and -Independent Manners
Source: PLoS One. 2014 Jan 13;9(1):e84807. doi: 10.1371/journal.pone.0084807 (PMC3890271; doi:10.1371/journal.pone.0084807)
Supplement: Table S1 — Top five ontology terms with molecular and cellular function of upregulated genes after DSF or 5-FU treatment. (DOC) [file pone.0084807.s009.doc]

**Table S1.** Top five ontology terms with molecular and cellular function of upregulated genes after DSF or 5-FU treatment

Treatment Rank Ontology terms p value

DSF 1 Cell death 5.38E-05

2 Post-translational modification 7.88E-04

3 Gene expression 8.36E-04

4 DNA replication, recombination, and repair 8.95E-04

5 Carbohydrate metabolism 9.25E-04

5-FU 1 Cellular response to therapeutics 8.52E-06

2 Cellular assembly and organization 1.34E-05

3 Cellular function and maintenance 2.04E-05

4 DNA replication, recombination, and repair 4.13E-05

5 Cell death 6.32E-05
