# Supplementary material for: Disulfiram Eradicates Tumor-Initiating Hepatocellular Carcinoma Cells in ROS-p38 MAPK Pathway-Dependent and -Independent Manners
Source: PLoS One. 2014 Jan 13;9(1):e84807. doi: 10.1371/journal.pone.0084807 (PMC3890271; doi:10.1371/journal.pone.0084807)
Supplement: Table S2 — Top five ontology terms with molecular and cellular function of downregulated genes after DSF or 5-FU treatment. (DOC) [file pone.0084807.s010.doc]

**Table S2.** Top five ontology terms with molecular and cellular function of downregulated genes after DSF or 5-FU treatment

Treatment Rank Ontology terms p value

DSF 1 Lipid metabolism 1.29E-12

1 Small molecule biochemistry 1.29E-12

1 Vitamin and mineral metabolism 1.29E-12

4 Amino acid metabolism 1.73E-06

5 Molecular transport 8.73E-06

5-FU 1 RNA post-transcriptional modification 2.64E-13

2 Gene expression 1.55E-09

3 Protein synthesis 2.81E-09

4 Carbohydrate metabolism 7.23E-05

4 Small molecule biochemistry 7.23E-05
