# Supplementary material for: Disulfiram Eradicates Tumor-Initiating Hepatocellular Carcinoma Cells in ROS-p38 MAPK Pathway-Dependent and -Independent Manners
Source: PLoS One. 2014 Jan 13;9(1):e84807. doi: 10.1371/journal.pone.0084807 (PMC3890271; doi:10.1371/journal.pone.0084807)
Supplement: Table S3 — Primer sequences used for real-time RT-PCR. (DOC) [file pone.0084807.s011.doc]

**Table S3.** Primer sequences used for real-time RT-PCR

Gene name Sequence

*GPC3* Forward 5'- CTCCGCTTCCTTGCAGAA -3’

Reverse 5'- TGCTTATCTCGTTGTCCTTCG -3’

*CDH1* Forward 5'- AACAGGAACACAGGAGTCATCA -3’

Reverse 5'- AGGTCAGCAGCTTGAACCAC -3’

*AFP* Forward 5'- ATGGCCATCACCAGAAAAAT -3’

Reverse 5'- CATAAGTGTCCGATAATAATGTCAGC -3’

*CDK2* Forward 5'- GAGTCCCTGTTCGTACTTACACC -3’

Reverse 5'- CCAGGCTCCAGATGTCCA -3’

*p57 KIP2* Forward 5'- TCCATCTCCCACATCTGGTT -3’

Reverse 5'- GCAGCTACAGCTTGTGAGTGA -3’

*GAPDH* Forward 5'- CTGACTTCAACAGCGACACC -3’

Reverse 5'- TAGCCAAATTCGTTGTCATACC -3’
